# Using Self‐Reported Training Characteristics to Better Understand Who Is More Likely to Sustain Running‐Related Injuries Than Others: The Garmin‐RUNSAFE Running Health Study

**DOI:** 10.1111/sms.70004

**Published:** 2024-12-23

**Authors:** Josefin Abrahamson, Ida Lindman, Mathilde Birksoe Eriksen, Albert Kibsgaard, Rasmus Oestergaard Nielsen

**Affiliations:** ^1^ Orthopaedic Research Unit Sahlgrenska University Hospital Gothenburg Sweden; ^2^ Department of Health and Rehabilitation, Institute of Neuroscience and Physiology at Sahlgrenska Academy University of Gothenburg Gothenburg Sweden; ^3^ General Practice/Family Medicine, School of Public Health and Community Medicine, Institute of Medicine, Sahlgrenska Academy University of Gothenburg Gothenburg Sweden; ^4^ Research, Education, Development & Innovation Primary Health Care, Region Västra Götaland Västra Götaland Sweden; ^5^ Department of Public Health Aarhus University Aarhus Denmark; ^6^ Research Unit for General Practice Aarhus Denmark

**Keywords:** injuries, running, running‐related injury, training characteristics

## Abstract

Running is a popular form of physical activity, yet it comes with risks, including running‐related injuries (RRIs). This cohort study aimed to use self‐reported baseline data on running experience, weekly running frequency, greatest running distance in 1 week, and running program to investigate if certain adult runners were more likely to sustain RRI than others. Runners, aged ≥ 18 years, familiar with the English language and using a Garmin watch to track their running were included. Running data and injury status were collected prospectively through the Garmin Connect application and weekly questionnaires over 18 months. Exposure variables were self‐reported running experience (years), weekly running frequency and distance, and use of structured running program (last 3 months before inclusion). The outcome was RRI. Time to event statistics was used to calculate cumulative risk differences (cRD) within groups of each exposure. Data were analyzed at 1000 km (km). A total of 7391 runners were included. The cumulative injury proportion was 57.8% [95% CI: 56.4%; 59.2%] after 1000 km. Those running > 105 km (cRD = −31.6, 95% CI −23.1; −40.1), 7 times per week (cRD = −47.1, 95% CI −35.9; −58.3) and followed a structured running program (cRD = 4.4; 95% CI 0.9; 7.8) had the fewest new RRIs. For running experience, those with few (< 1 years) or many years (> 40 years) of experience had the most RRIs. Runners were more prone to sustain an RRI if they had few (< 1) or many (> 40) years of running experience, lower total weekly running frequency (< 2 times/week), shorter weekly running distance (< 25 km), or did not use a structured running program.

## 1 Introduction

Running has become one of the most popular forms of physical activity, both at recreational and competitive levels. The popularity of running may be due to its easy accessibility and simple execution, and its positive impacts on physical health [1]. Running has been suggested to have beneficial effects on body mass, body fat, VO_2_‐max, resting heart rate, triglycerides, HDL cholesterol and mental health, and reduce the risk of all‐cause and cardiovascular mortality [2, 3]. The longer the training period, the larger the achieved health benefits [1]. In contrast to the positive aspects of running, running‐related injuries (RRI) are common and affect millions of people every year, making it a considerable public health issue [4]. Studies have reported a wide variation of injury incidence proportion in runners ranging from 3%–85% [4]. The considerable range is likely due to different study designs, injury definitions, and length of follow‐up. Furthermore, RRIs represent a prominent barrier to continue running and is considered the most common reason for quit running across various running disciplines and experience levels [5, 6]. To ensure adherence to running, it is important to better understand who is more likely to sustain an RRI.

It is commonly believed that factors like age, BMI, sex, health factors (i.e., previous injuries, smoking, and co‐morbidities), running experience, shoe type, and training characteristics (e.g., duration, frequency, distance, speed) are associated with an increased risk of RRIs. While previous injury and higher BMI have been shown to increase the risk of an RRI [7], conflicting results, regarding significant associations for the other factors, have been reported [8, 9, 10, 11]. This may be due to differences in study population, injury definitions, runner types (elite or recreational), running distances, and the follow‐up period [4]. Still, it remains uncertain whether runners with a certain previous running experience, weekly running frequency (running sessions) or average weekly running distance are more or less likely to sustain an injury than other runners. The same line of reasoning applies to the use of structured programs for running. Possibly, runners using a structured running programs may be less likely to sustain an RRI compared with those not using a running program.

The aim of the study was to use self‐reported baseline data on running experience, weekly running frequency, greatest running distance in 1 week and running program to investigate if certain adult runners were more likely to sustain a running‐related injury than others. The hypothesis of this study was that runners with shorter running experience, the lowest and highest running frequency and distance, and not using a structured training program, were more likely to sustain a running‐related injury.

## 2 Materials and Methods

### 2.1 Study Design

This study was part of the prospective cohort study “The Garmin‐RUNSAFE Running Health Study” [12]. The Garmin‐RUNSAFE Running Health Study aimed to evaluate the relationship between changes in training‐load and the occurrence of RRI, and subsequently how an RRI was associated with other variables. The study was an 18‐month prospective cohort study including runners of all levels around the world. The study followed the Strengthening the Reporting of Observational studies of Epidemiology (STROBE) checklist [13] (Data S1).

### 2.2 Study Population

Adult runners from all around the world using a Garmin wearable device while tracking their training were recruited for this study between July 4 and December 1, 2019. Runners who met the following criteria were included: (1) Runners ≥ 18 years of age; (2) using a Garmin GPS wearable device and uploading their running data via the Garmin Connect application (app), which is a worldwide web‐based training diary (https://connect.garmin.com/); (3) familiar with the English language. No baseline exclusion criteria were applied, however, participants were excluded if they stopped uploading their running sessions to the Garmin Connect app, if multiple people used the same Garmin Connect profile, or if they discontinued answering the weekly questionnaires as described below. The follow‐up extended from August 2019 to December 2020, and, therefore, the duration of the follow‐up period ranged up to 18 months among participants.

### 2.3 Ethics

All participants were required to sign an online consent form prior to inclusion in the study. The study was approved by the Ethics committee of central Denmark Region (request number: 22/2016; record number: 1‐10‐72‐189‐16). The study has received authorization from the Danish Data Protections agency and its data collection procedures and storage of data. In accordance with the Danish Act on Research and Health Projects (section 14, no. 2), this study does not need permission from the system of research ethics committees as it falls under the category of an observational study.

### 2.4 Data Collection

Data were collected objectively via the participant's Garmin watches and Garmin Connect profiles, and subjectively via baseline and weekly questionnaire throughout the follow‐up. Objective data used in this study was the running distance, which was collected by global positioning system (GPS). The validity of running distance with the use of GPS‐based quantification has shown to be higher compared with subjective self‐reporting [14, 15].

The baseline questionnaire was completed at inclusion. This included questions regarding running history (i.e., greatest running distance in 1 week, average running frequency per week, and the use of a structured running program) last 3 months before inclusion, demographics and running experience in years. Every Sunday, a weekly questionnaire including injury status was automatically e‐mailed to the runners. By the question “In the past week, have you had a musculoskeletal injury, or have you experienced a problem to muscles, tendons or bones that is fully or partly caused by running?”, the runners classified themselves as (1) injury‐free; (2) uninjured, yet with problems (i.e., new or same problems as reported last week); (3) injured. An injury was defined as something painful and irritating, leading to a reduction in running activity (i.e., volume, frequency, intensity), whereas a problem was defined as less severe than an injury, causing pain and irritation, yet allowed the runners to maintain their running activity. This methodology aligns with the running injury consensus outlined by the Oslo Trauma Research Centre Questionnaire [16] and Yamato et al. [17]. The main outcome was RRI, which was dichotomized into two groups: (1) injured, and (2) uninjured. The category ‘uninjured with problems’ was defined as ‘uninjured’ meaning that only a reduction in running activity was equivalent to an injury.

The four exposure/predictors of interest were derived from the baseline questionnaire: (i) running experience in years; (ii) greatest running distance in 1 week; (iii) running frequency (times per week), and (iv) to follow or not to follow a structured running program. Running experience in years was a categorized variable based on the question: “For how many years have you been a runner (years of running experience)?”. Greatest weekly running distance in kilometer was reported following: “What is your greatest total weekly running distance in the PAST THREE MONTHS?”, whereas frequency (number of running sessions) was based on: “How many times per week, have you typically been running on average in the PAST THREE MONTHS?”. Finally, running program was based on the question: “How do you structure your running?”. An overview of the questions and the corresponding response categories is presented in Data S2. All questions were inserted as radio‐buttons in the questionnaire enabling each runner to select one (not multiple) response option. In Nielsen et al., all questionnaires distributed to the runners are found in the Supporting Information material [12].

### 2.5 Statistics

Time‐to‐event analysis was used to estimate the cumulative incidence proportion (CIP) of injuries for each exposure group within the four exposures (running experience, total weekly running distance and frequency last 3 months before inclusion, and the use of structured running program) [18]. To calculate the CIP as the proportion of injury‐free athletes while applying the concept of censored observations, one can use the following equation (survival function—the readers may think of ‘survival’ as remaining ‘uninjured’ in sports medicine): *S*(*t*) = pr(*T* > *t*), where *S*(*t*) is the survival at time *t*, *T* is the time to injury and pr is the probability that a participant will ‘survive’ (remain uninjured) beyond time *t*. Consequently, the CIP is not merely a result of dividing the number of injuries by the runners at risk, since kilometers‐to‐injury or censoring is incorporated into the equation [19]. The pseudo‐observation method was used to calculate the cumulative risk difference (cRD), which was determined by subtracting the CIP of the different exposure categories from the reference group, with the lowest exposure category used as the reference group. For the dichotomized variable “use/no use of a structured running program”, the reference group was ‘yes/use’. The time point 1000 km was chosen for the main analyses, since this has been reported in previous studies [20, 21], and was chosen prior to any of the data processing. As other time point could have been used, a Kaplan–Meier graph is presented visualizing the proportion of injury‐free runners of the different exposure groups at other time points as well and sensitivity analyses at 500 km and 1500 km are presented in the Data S3. Runners were censored from the study if they stopped uploading their running sessions for a period of 6 months, ceased responding to the weekly questionnaires for four consecutive weeks, or expressed unwillingness to continue participating. If runners did not upload data on running activity within a 6‐month period and/or on injury status within a 3‐week period, it was assumed that they did not run and/or that they did not sustain an injury. Confidence interval (CI) and *p*‐value were calculated to investigate whether there was a statistically significant difference between the exposure categories in the pseudo‐observations. Data management and analyses were performed in STATA (version 18, Texas, USA). To calculate the cRD and cumulative relative risk, a generalized linear model using robust variance estimation was used including a Wald‐test to test for difference between groups [22]. For this, the stpsurv‐package in STATA was used [23]. For test of differences between the CIPs of two exposure groups, a *p* < 0.05 was considered as statistically significant. All p‐values and CIs were derived directly from the STATA outputs.

## 3 Results

**FIGURE 1:**
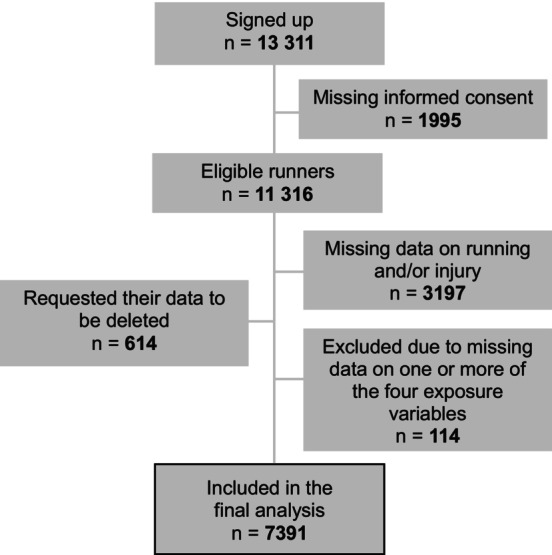
Flowchart of included runners. Abbreviation: RRI, running‐related injury.

A total of 13 311 runners completed the enrolment questionnaire, of which 7391 runners (77.6% male) were included in the present study (Figure 1). The majority had between 5–10 (26.1%), 10–20 (22.6%) or 20–40 (19.1%) years of running experience, were running two (17.9%), three (33.3%) or four (20.0%) times per week, and had a total running distance in 1 week of 15–25 (17.2%), 25–35 (17.0%) or 35–45 (14.9%) kilometers. In total, 5601 (75.8%) followed a structured running program. A total of 3726 (50.4%) of the runners sustained at least one RRI during follow‐up. Table 1 presents the demographic data.

**TABLE 1:** Demographic data of included runners.

Variable	Numbers
No. of runners, *n*	7391
Male, *n* (%)	5735 (77.6)
Age, years, mean (SE)	46.1 (0.13)
BMI, kg/m^2^, mean (SE)^a^	24.3 (0.04)
New RRI up to 1.000 km of running	
Yes, *n* (%)	3250 (44.0)
No, *n* (%)	4141 (56.0)
Continent, *n* (%)	
Europe	4164 (56.3)
North America	2982 (40.4)
Africa	138 (1.9)
Asia	52 (0.7)
South America	39 (0.5)
Oceania	16 (0.2)
Running experience, *n* (%)	
Below 1 year	210 (2.8)
1–3 years	816 (11.0)
3–5 years	959 (13.0)
5–10 years	1927 (26.1)
10–20 years	1669 (22.6)
Over 40 years	363 (4.9)
Do not know	35 (0.5)
Running frequency (times/week), *n* (%)	
1 time or less	651 (8.8)
2 times	1323 (17.9)
3 times	2459 (33.3)
4 times	1478 (20.0)
5 times	824 (11.1)
6 times	350 (4.7)
7 times	137 (1.9)
Over 7 times	66 (0.9)
Do not know	103 (1.4)
Running distance (in 1 week), *n* (%)	
< 15 km	862 (11.7)
15–25 km	12 (17.2)
25–35 km	1258 (17.0)
35–45 km	1104 (14.9)
45–55 km	903 (12.2)
55–65 km	585 (7.9)
65–75 km	401 (5.4)
75–85 km	291 (3.9)
85–95 km	177 (2.4)
95–105 km	150 (2.0)
> 105 km	256 (3.5)
Do not know	134 (1.8)
Follow a structured running program, *n* (%)	
Yes	5601 (75.8)
No	1575 (21.3)
Other	215 (2.9)
Fastest 5 km run, min, *n* (%)^b^	
< 14	15 (0.2)
14–17	111 (1.5)
17–20	736 (10.0)
20–23	1684 (22.8)
23–26	1905 (25.8)
26–29	1230 (16.6)
29–32	710 (9.6)
32–35	386 (5.2)
> 35	381 (5.2)
Do not know	232 (3.1)
Main reason for running, *n* (%)	
Well‐being/health	4236 (57.4)
To get fitter	1003 (13.6)
To compete	984 (13.3)
To get a break from work/family	339 (4.6)
To lose weight	305 (4.1)
As supplementary training	297 (4.0)
To socialize	43 (0.6)
Other	151 (2.0)
Do not know	33 (0.4)

Abbreviations: BMI, body mass index; kg, kilogram; km, kilometer; m, meters; min, minutes; n, numbers; SE, standard error.

^a^ BMI is based on 6886 runners due to missing responses (*n* = 505) regarding BMI.

^b^ Based on 7390 runners due to missing value (*n* = 1).

**FIGURE 2:**
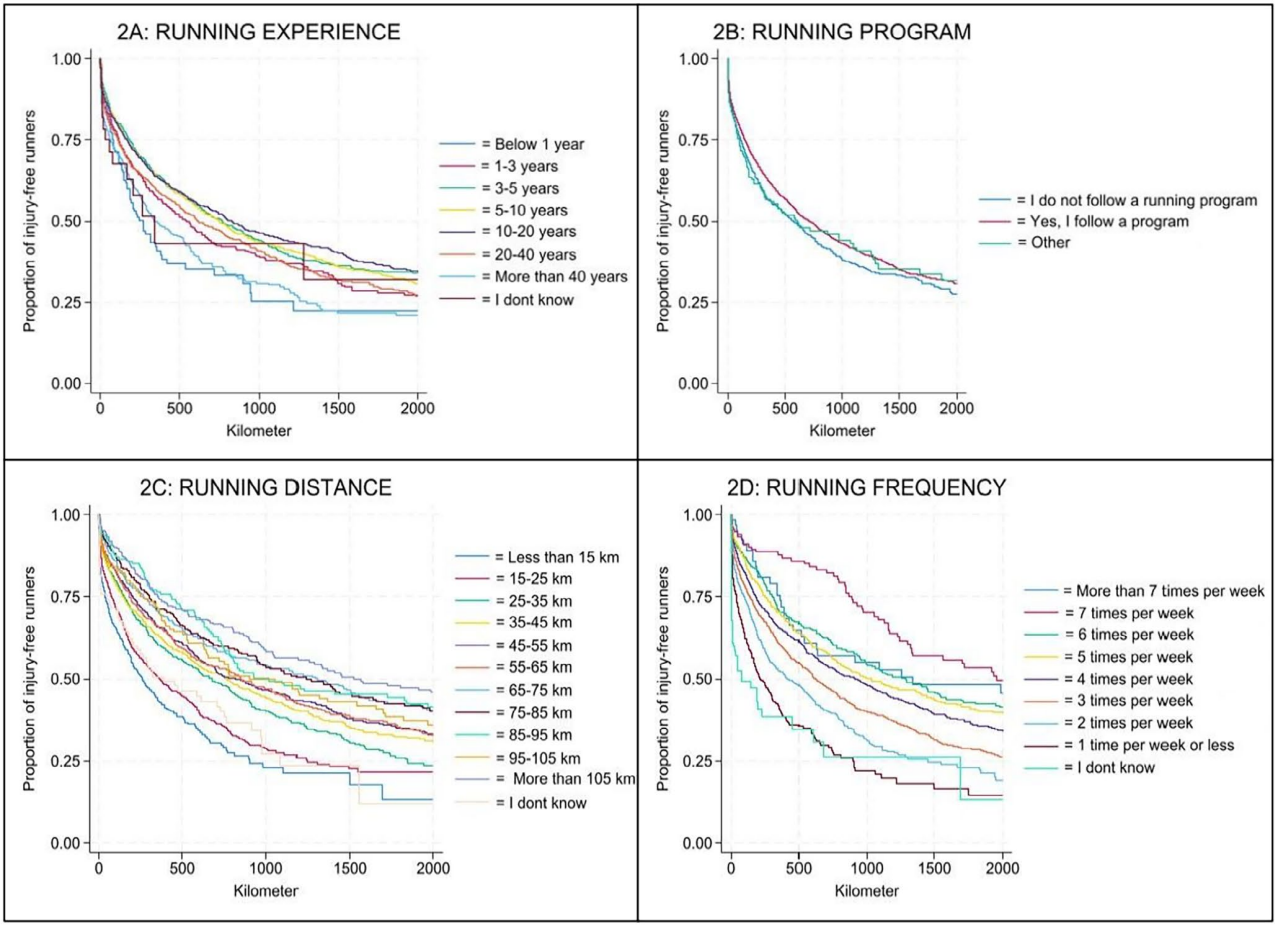
(A–D) Kaplan–Meier graphs for the proportion of injury‐free runners using kilometers as timescale on the *x*‐axis. Figure 2A displays running experience in years, figure 2B the use of structured running program at baseline, figure 2C displays the greatest running distance (km, kilometer) in 1 week during the last 3 months before baseline, and figure 2D displays average weekly running frequency (times per week) during the last 3 months before baseline.

When taking censoring into account, the CIP at 1000 km was 57.8% [95% CI: 56.4%; 59.2%]. Table 2 presents the main results including the CIP for each exposure group and the cRD within each of the four exposure variables at 1000 km. Figure 2 displays Kaplan–Meier graphs of the proportion of injury‐free runners at given distances of running, by exposure groups.

**TABLE 2:** Proportion of runners sustaining a running‐related injury up to 1000 km of running among 7391 runners with different running experiences, running frequency and distance, and the use or no use of a structured running program 3 months preceding baseline including the absolute (cRD) and relative (cRR) difference between the exposure groups.

	Yes, RRI, *n* = 3250	No, RRI, *n* = 4141	CIP, %	cRD [95% CI], %‐points	cRR [95% CI], %
Running experience					
Below 1 year	93	117	69.1	0 (ref.)	1 (ref.)
1–3 years	351	465	60.7	−8.4 [−17.7; 0.1]	0.88 [0.76; 1.01]
3–5 years	388	571	56.0	−13.1 [−22.2; −3.9]**	0.81 [0.71; 0.93]**
5–10 years	825	1102	54.5	−12.7 [−21.4; −4.0]**	0.82 [0.72; 0.93]**
10–20 years	697	972	53.5	−15.6 [−24.4; −6.7]**	0.78 [0.68; 0.88]***
20–40 years	673	739	59.2	−9.9 [−18.8; −1.0]*	0.86 [0.75; 0.98]*
Over 40 years	209	154	69.0	−0.1 [−9.5; 11.3]	1.01 [0.59; 1.23]
I do not know	14	21	58.7	−10.4 [−32.3; 11.6]	0.85 [0.59; 1.22]
Running frequency (times/week)					
1 time or less	310	341	71.8	0 (ref.)	1 (ref.)
2 times	618	705	66.8	−5.0 [−10.8; 0.7]	0.93 [0.86; 1.01]
3 times	1106	1353	59.9	−11.9 [−17.1; −6.6]***	0.83 [0.77; 0.90]***
4 times	626	852	51.4	−20.4 [−26.1; −14.8]***	0.71 [0.65; 0.78]***
5 times	339	485	48.8	−23.0 [−29.3; −16.8]***	0.68 [0.61; 0.76]***
6 times	137	213	43.3	−28.5 [−36.4; −20.6]***	0.60 [0.51; 0.71]***
7 times	34	103	24.7	−47.1 [−58.3; −35.9]***	0.34 [0.23; 0.52]***
Over 7 times	26	40	41.7	−30.1 [−44.5; −14.7]***	0.58 [0.41; 0.83]***
I do not know	54	49	72.7	0.9 [−11.8; 13.5]	1.01 [0.85; 1.20]
Running distance (in 1 week)					
< 15 km	372	490	69.4	0 (ref.)	1 (ref.)
15–25 km	606	664	68.5	−0.9 [−6.2; 4.3]	0.99 [0.91; 1.06]
25–35 km	547	711	59.5	−9.9 [−15.2; −4.7]***	0.86 [0.79; 0.93]***
35–45 km	503	601	55.6	−13.8 [−19.3; −8.4]***	0.80 [0.73; 0.87]***
45–55 km	389	514	53.2	−16.2 [−21.9; −10.5]***	0.77 [0.73; 0.87]***
55–65 km	263	322	52.4	−17.0 [−23.4; −10.6]***	0.75 [0.68; 0.84]***
65–75 km	154	247	43.8	−25.6 [−32.8; −18.4]***	0.63 [0.54; 0.73]***
75–85 km	115	176	43.7	−25.7 [−33.8; −17.6]***	0.63 [0.53; 0.75]***
85–95 km	75	102	50.0	−19.4 [−29.2; −9.5]***	0.72 [0.60; 0.87]***
95–105 km	64	86	48.6	−20.8 [−31.3; −10.2]**	0.70 [0.57; 0.86]***
> 105 km	94	162	37.8	−31.6 [−40.1; −23.1]***	0.54 [0.44; 0.67]***
I do not know	68	66	68.5	0.9 [−10.2; 12.0]	1.01 [0.86; 1.19]
Running program					
Yes	2483	3118	56.8	0 (ref.)	1 (ref.)
No	668	907	61.2	4.4 [0.9; 7.8]*	1.08 [1.02; 1.14]*
Other	99	116	55.9	−0.9 [−9.3; 7.5]	0.98 [0.85; 1.14]

Abbreviations: CI, confidence interval in percentage; CIP, cumulative incidence proportion at 1000 km; cRD, cumulative risk difference (the difference in percent‐point compared with the reference group); cRR, cumulative relative risk; km, kilometer; ref., reference; RRI, running‐related injury.

* Statistical difference at *p* < 0.05.

** Statistical difference at *p* < 0.01.

*** Statistical difference at *p* < 0.001.

## 4 Discussion

This study aimed to investigate if years of running experience, total weekly running frequency, maximum weekly running distance, or the use a structured running program predispose runners of sustaining RRIs. The main findings of the study were that total weekly running distance and frequency last 3 months before inclusion had an almost linear pattern with new RRIs, in the sense that most injuries occurred among those with least running frequency (1 time per week or less) and running distance (less than 15 km per week) and fewest new injuries occurred among those running more than 7 times per week or ran more than 105 km per week. In contrast, running experience had a U‐shaped relationship as those with lowest and highest experience sustained most injuries.

The cumulative injury incidence at 1000 km in this study was 57.8% [95% CI: 56.4%; 59.2%] across all runners. Previous similar studies have shown an incidence proportion of 30% [10, 24, 25]. However, due to different study designs and definition of injuries, other studies have estimated injury proportions between 3%–85% [4].

Runners with the shortest and longest running experience had, in this study, the highest proportion of RRIs (69% in both groups). Running experience had a U‐shaped relationship with RRIs, as the proportion of RRIs decreased to its lowest point of 54% at 10–20 years of running experience, and then started to increase again. This U‐shaped relationship is in contrast with a previous study by Poppel et al. [8], where the odds ratio was 1.77 (significant) for those with less than 5 years of running experience and 1.19 (non‐significant) for those between 5 and 10 years, when comparing with runners with more than 10 years of experience. However, they had an endpoint of > 10 years, while this study had > 40 years, thus it is unknown if the U‐shaped relationship would be true if that study instead had used an endpoint of > 40 years.

New RRIs in relation to the maximum weekly total amount of running last 3 months before inclusion followed a slightly decreasing exponential relationship. For running frequency, the proportion of RRIs decreased from 72% in those running once a week or less, to 25% in those running seven times a week. Although, a slight increase could be seen (42%) in the group who ran more than seven times a week. The proportion of RRIs, for running distance, decreased from 69% in runners with less than 15 kilometers in 1 week, to 38% in runners with more than 105 km in 1 week. Joints, muscles, and tendons need to be trained specifically for the demands of running. Proper training leads to biological adaptations that increase the body's ability to tolerate mechanical stress. This improved resilience helps protect runners from injuries [26]. This might explain why the proportion of RRIs decreased in this study with more frequency and distance. On the other hand, lack of sufficient recovery is a risk factor for injuries in general [26]. With more running sessions a week, including running frequency and maximum running distance, there is less time for recovery, thus increasing the risk of RRI. This could partly explain the increased proportion of RRIs in the group running more than seven times a week in this study.

Runners not following a structured running program sustained more RRIs (61%) compared with those who did (57%, *p* = 0.023). This is in line with a previous study where a lower incidence of RRI was found among runners following any type of plan or program [8, 27]. Excessively steep and/or rapid increase in workload, particularly large weekly changes in workload (intensity, frequency, and duration) have been shown to significantly increase the risk of RRI [26]. Following a structured program allows for better monitoring of workload, which can help runners understand how their bodies are responding to training, recognize signs of fatigue, and assess recovery needs. By making necessary adjustments to the program, runners may reduce their risk of injury.

### 4.1 Strength and Limitations

Strengths of this study include the prospective cohort study design, a large sample size, the use of time‐to‐event statistics to calculate CIP and cRD and the long follow‐up period of 18 months. Although, some of the exposure groups had a small number of participants, resulting in a larger 95% CI compared with other exposure groups of the same variable. The use of an absolute measure of association in the data analysis is a further strength. This study used cRD, presenting the absolute cumulative difference in the proportion of runners sustaining an RRI, allowing runners, coaches, and clinicians to evaluate whether the differences in sustained injuries were relevant [28]. Another strength is the quantification of running data throughout the follow‐up period measuring the distance until possibly sustaining an RRI or censoring. Despite these strengths, there are limitations needed to be addressed. Firstly, the outcome (RRI) was self‐reported by the runners by weekly questionnaires, thus dependent on each runner's pain perception and understanding of the definition of injury. Secondly, there may also be a risk for “delayed” injury reporting or that an injury might not be reported at all. This could occur if the runner fails to complete the weekly injury questionnaire for a couple of weeks (and subsequently recovers before starting to answer the weekly injury questionnaire again) or fail to upload their running data for a period shorter than 6 months. However, to assess the extent of missing data, a thorough review of the dataset showed that the amount of such missing data was minimal and did not significantly affect the overall integrity of the results. Lastly, there is a risk of recall bias when runners answer questions regarding experience and their running status last 3 months before inclusion. Although this study included adult runner from all over the world (limited to English‐speaking) at all types of running levels, the majority were male (78%) and from Europe (56%) or North America (40%). Therefore, the results of this study may be limited for generalization to females and other continents than Europe and North America. Another potential limitation is the way the data on the exposures were collected since the runners were asked to report data on an ordinal scale, not a continuous scale. Such approach may lead to information problems and bias [29].

## 5 Conclusion

Sustaining a running‐related injury was more frequent among runners with less than 1 year or over 40 years of running experience, runners with low weekly running frequency (≤ once a week), runners with shorter total distance per week (< 25 km), and for runners not following a structured running program.

## 6 Perspective

By analyzing data from 7391 adult runners from across the world, this study provides comprehensive understanding of who is more likely to sustain RRIs based on previous training‐related characteristics. The findings of this study showed that subgroups of runners may be more susceptible to RRIs than others, especially those with few (< 1) or many (> 40) years of running experience, those with a weekly running frequency of 1 or less running session per week, those who have a weekly running distance of less than 25 km and those who did not use a structured running program. This study is a part of the Garmin‐RUNSAFE Running Health study which aims to evaluate the relationship between changes in training‐load and the occurrence of RRI, and subsequently how RRI is associated with other variables [12]. The result of this study is another piece in the puzzle to gain a better understanding of target populations for RRIs interventions, along with previous results including those from the Garmin‐RUNSAFE Running Health study [30]. The findings of this study empower sports medicine practitioners to extract knowledge concerning what characterizes the runners who sustained the most RRIs.

## Ethics Statement

The study was approved by the Ethics committee of central Denmark Region (request number: 22/2016. Record number 1‐10‐72‐189‐16).

## Consent

An online informed consent form was signed by all included runners.

## Conflicts of Interest

The authors declare no conflicts of interest.

## Supporting information


Data S1.

Data S2.

Data S3.

## Data Availability

The data are not publicly available due to privacy or ethical restrictions.
