# Supplementary material for: Using Self‐Reported Training Characteristics to Better Understand Who Is More Likely to Sustain Running‐Related Injuries Than Others: The Garmin‐RUNSAFE Running Health Study
Source: Scand J Med Sci Sports. 2024 Dec 23;35(1):e70004. doi: 10.1111/sms.70004 (PMC11664494; doi:10.1111/sms.70004)
Supplement: Supplementary file 2 — Data S2. [file SMS-35-e70004-s002.docx]

**Supplementary Material S2:**

Wording of the questions underpinning the four exposures/predictors used in the present study.

**RUNNING EXPERIENCE**

Was based on question 2 in the baseline questionnaire:

***For how many years have you been a runner (years of running experience)?***

- Below 1
- 1-2
- 2-3
- … [continued]
- 35-40
- More than 40
- I don´t know

**RUNNING FREQUENCY**

Was based on question 7 in the baseline questionnaire:

***How many times per week, have you typically been running on average in the PAST THREE MONTHS?***

- - More than 7 times per week
  - 7 times per week
  - 6 times per week
  - 5 times per week
  - 4 times per week
  - 3 times per week
  - 2 times per week
  - 1 time per week
  - Less than one time per week
  - I have not been running at all during the past three months
  - I don´t know

**RUNNING DISTANCE**

Was based on question 7 in the baseline questionnaire:

***What is your greatest total weekly running distance in the PAST THREE MONTHS?***

- Less than 5 km in one week / less than 3.2 miles in one week
- Between 5-15 km in one week / between 3.2-9.6 miles in one week
- Between 15-25 km in one week / between 9.6-15.9 miles in one week
- Between 25-35 km in one week / between 15.9-21.1 miles in one week
- Between 35-45 km in one week / between 21.1-27.2 miles in one week
- Between 45-55 km in one week / between 27.2-34.3 miles in one week
- Between 55-65 km in one week / between 34.3-40.7 miles in one week
- Between 65-75 km in one week / between 40.7-46.1 miles in one week
- Between 75-85 km in one week / between 46.1-52.1 miles in one week
- Between 85-95 km in one week / between 52.1-59.5 miles in one week
- Between 95-105 km in one week / between 59.5-65.4 miles in one week
- More than 105 km in one week / more than 65.4 miles in one week
- I don´t know

**RUNNING PROGRAM**

Was based on question 11 in the baseline questionnaire:

***How do you structure your running?***

- I do not follow a running program / I do not structure my running schedule
- I follow a training program from a coach
- I follow a training program from a newspaper, magazine or the internet
- I follow a training program from a friend or relative
- Self-organized – I schedule my running on my own
- Other
