# Supplementary material for: Using Self‐Reported Training Characteristics to Better Understand Who Is More Likely to Sustain Running‐Related Injuries Than Others: The Garmin‐RUNSAFE Running Health Study
Source: Scand J Med Sci Sports. 2024 Dec 23;35(1):e70004. doi: 10.1111/sms.70004 (PMC11664494; doi:10.1111/sms.70004)
Supplement: Supplementary file 3 — Data S3. [file SMS-35-e70004-s003.docx]

**Supplementary material**

Sensitivity analyses at 500 and 1500 kilometers of running.

**500 kilometers**

Proportion of runners sustaining a running-related injury up to 500 kilometers of running among 7391 runners with different running experiences, running frequency and distance, and the use or no use of a structured running program three months preceding baseline including the absolute (cRD) and relative (cRR) difference between the exposure groups.

|  |  |  | **CIP**  **%** | **cRD [95% CI]**  **%-points** | **cRR [95% CI]**  **%** |
| --- | --- | --- | --- | --- | --- |
| **Running experience** |  |  |  |  |  |
| Below 1 year |  |  | 58.0 | 0 (ref.) | 1 (ref.) |
| 1-3 years |  |  | 48.1 | -9.9 [-18.4; -1.4]* | 0.82 [0.71; 0.96]* |
| 3-5 years |  |  | 40.9 | -17.1 [-25.5; -8.7]** | 0.70 [0.60; 0.82]** |
| 5-10 years |  |  | 41.6 | -16.4 [-24.4; -8.4]** | 0.71 [0.61; 0.82]** |
| 10-20 years |  |  | 41.1 | -16.9 [-25.0; -8.9]** | 0.70 [0.61; 0.81]*** |
| 20-40 years |  |  | 45.6 | -12.4 [-20.5; -4.3]* | 0.78 [0.67; 0.90]* |
| Over 40 years |  |  | 55.3 | -2.7 [-12.3; 6.7] | 0.95 [0.80; 1.12] |
| I do not know |  |  | 54.2 | -3.8 [-23.9; 16.2] | 0.93 [0.84; 1.34] |
| **Running frequency (times/week)** | | | | |  |
| 1 time or less |  |  | 59.8 | 0 (ref.) | 1 (ref.) |
| 2 times |  |  | 51.4 | -8.4 [-13.6; -3.2]*** | 0.85 [0.78; 0.94]*** |
| 3 times |  |  | 45.4 | -14.4 [-19.2; -9.6]*** | 0.75 [0.69; 0.82]*** |
| 4 times |  |  | 38.0 | -21.8 [-26.9; -16.6]*** | 0.63 [0.57; 0.70]*** |
| 5 times |  |  | 35.6 | -24.2 [-29.9; -18.5]*** | 0.59 [0.52; 0.67]*** |
| 6 times |  |  | 31.6 | -28.2 [-35.4; -21.0]*** | 0.52 [0.43; 0.64]*** |
| 7 times |  |  | 10.4 | -49.4 [-59.7; -39.2]*** | 0.17 [0.07; 0.42]*** |
| Over 7 times |  |  | 35.2 | -24.6 [-38.7; -10.5]*** | 0.58 [0.39; 0.86]*** |
| I do not know |  |  | 63.5 | 3.7 [-7.7; 15.3] | 1.06 [0.88; 1.27] |
| **Running distance (in one week)** | | | | |  |
| < 15km |  |  | 57.0 | 0 (ref.) | 1 (ref.) |
| 15-25km |  |  | 54.0 | -3.0 [-7.8; 1.8] | 0.94 [0.86; 1.03] |
| 25-35km |  |  | 44.3 | -12.7 [-17.5; -7.8]*** | 0.77 [0.70; 0.85]*** |
| 35-45km |  |  | 41.6 | -15.4 [-20.3; -10.4]*** | 0.73 [0.65; 0.80]*** |
| 45-55km |  |  | 39.0 | -18.0 [-23.5; -13.1]*** | 0.68 [0.60; 0.76]*** |
| 55-65km |  |  | 40.7 | -16.3 [-22.1; -10.4]*** | 0.71 [0.72; 0.81]*** |
| 65-75km |  |  | 33.8 | -23.2 [-29.8; -16.6]*** | 0.59 [0.49; 0.70]*** |
| 75-85km |  |  | 32.5 | -24.5 [-31.9; -7.1]*** | 0.57 [0.46; 0.70]*** |
| 85-95km |  |  | 27.3 | -29.7 [-38.7; -20.9]*** | 0.47 [0.35; 0.65]*** |
| 95-105km |  |  | 34.6 | -22.4 [-32.0; -12.7]** | 0.60 [0.46; 0.79]*** |
| >105km |  |  | 27.6 | -29.4 [-37.2; -21.6]*** | 0.48 [0.37; 0.62]*** |
| I do not know |  |  | 53.0 | -4.0 [-14.1; 6.1] | 0.92 [0.76; 1.12] |
| **Running program** |  |  |  |  |  |
| Yes |  |  | 42.9 | 0 (ref.) | 1 (ref.) |
| No |  |  | 47.3 | 4.4 [1.2; 7.5]*** | 1.10 [1.03; 1.18]** |
| Other |  |  | 47.6 | 4.7 [-2.9; 12.3] | 1.11 [0.94; 1.30] |
| Abbreviations: RRI, running-related injury; ref., reference; CIP, cumulative incidence proportion at 500 kilometers; cRD, cumulative risk difference (the difference in percent-point compared with the reference group); cRR, cumulative relative risk; CI, confidence interval in percentage; km, kilometer. * = statistical difference at p-value <0.05; ** = statistical difference at p-value <0.01; ***=statistical difference at p-value <0.001 | | | | | |

**1500 kilometers**

Proportion of runners sustaining a running-related injury up to 1500 kilometers of running among 7391 runners with different running experiences, running frequency and distance, and the use or no use of a structured running program three months preceding baseline including the absolute (cRD) and relative (cRR) difference between the exposure groups.

|  |  |  | **CIP**  **%** | **cRD [95% CI]**  **%-points** | **cRR [95% CI]**  **%** |
| --- | --- | --- | --- | --- | --- |
| **Running experience** |  |  |  |  |  |
| Below 1 year |  |  | 74.0 | 0 (ref.) | 1 (ref.) |
| 1-3 years |  |  | 68.7 | -5.3 [-15.1; 4.4] | 0.92 [0.81; 1.06] |
| 3-5 years |  |  | 63.9 | -10.1 [-19.7; -0.4]* | 0.86 [0.76; 0.99]* |
| 5-10 years |  |  | 64.0 | -10.0 [-19.2; -0.1]* | 0.86 [0.76; 0.98]* |
| 10-20 years |  |  | 59.4 | -14.6 [-23.8; -5.3]** | 0.80 [0.71; 0.91]*** |
| 20-40 years |  |  | 68.2 | -5.8 [-15.1; 3.5] | 0.92 [0.81; 1.05] |
| Over 40 years |  |  | 79.3 | 5.3 [-5.6; 16.2] | 1.07 [0.92; 1.23] |
| I do not know |  |  | 67.9 | -6.1 [-29.1; 16.9] | 0.91 [0.66; 1.28] |
| **Running frequency (times/week)** | | | | |  |
| 1 time or less |  |  | 77.3 | 0 (ref.) | 1 (ref.) |
| 2 times |  |  | 73.4 | -3.9 [-9.9; 2.1] | 0.95 [0.87; 1.02] |
| 3 times |  |  | 68.2 | -9.1 [-14.6; -3.6]*** | 0.88 [0.82; 0.95]*** |
| 4 times |  |  | 60.2 | -17.1 [-22.9; -11.2]*** | 0.78 [0.72; 0.85]*** |
| 5 times |  |  | 54.0 | -23.3 [-29.8; -16.7]*** | 0.70 [0.61; 0.77]*** |
| 6 times |  |  | 51.0 | -26.3 [-34.6; -18.0]*** | 0.66 [0.57; 0.76]*** |
| 7 times |  |  | 38.9 | -38.4 [-50.2; -26.6]*** | 0.50 [0.38; 0.67]*** |
| Over 7 times |  |  | 46.8 | -30.5 [-46.7; -14.3]*** | 0.61 [0.43; 0.84]*** |
| I do not know |  |  | 78.4 | 1.1 [-14.4; 12.2] | 0.98 [0.83; 1.17] |
| **Running distance (in one week)** | | | | |  |
| < 15km |  |  | 74.9 | 0 (ref.) | 1 (ref.) |
| 15-25km |  |  | 74.8 | -0.1 [-5.9; 5.2] | 0.99 [0.92; 1.07] |
| 25-35km |  |  | 68.6 | -6.3 [-11.9; -0.7]* | 0.92 [0.84; 0.99]* |
| 35-45km |  |  | 64.7 | -10.2 [-15.9; -4.5]*** | 0.86 [0.79; 0.93]*** |
| 45-55km |  |  | 61.9 | -13.0 [-18.9; -7.0]*** | 0.82 [0.76; 0.90]*** |
| 55-65km |  |  | 60.9 | -14.0 [-20.7; -7.3]*** | 0.81 [0.73; 0.90]*** |
| 65-75km |  |  | 50.6 | -24.3 [-31.9; -16.7]*** | 0.67 [0.59; 0.77]*** |
| 75-85km |  |  | 52.2 | -22.7 [-31.2; -14.2]*** | 0.69 [0.60; 0.81]*** |
| 85-95km |  |  | 51.7 | -23.2 [-33.6; -12.9]*** | 0.69 [0.57; 0.84]*** |
| 95-105km |  |  | 54.0 | -20.9 [-32.0; -9.8]** | 0.72 [0.59; 0.88]*** |
| >105km |  |  | 44.5 | -30.4 [-39.3; -21.4]*** | 0.59 [0.49; 0.72]*** |
| I do not know |  |  | 75.4 | 0.5 [-11.1; 12.2] | 1.01 [0.86; 1.17] |
| **Running program** |  |  |  |  |  |
| Yes |  |  | 64.9 | 0 (ref.) | 1 (ref.) |
| No |  |  | 66.9 | 2.0 [-1.5; 5.6] | 1.03 [0.98; 1.09] |
| Other |  |  | 64.8 | -0.1 [-9.0; 8.6] | 0.99 [0.87; 1.14] |
| Abbreviations: RRI, running-related injury; ref., reference; CIP, cumulative incidence proportion at 500 kilometers; cRD, cumulative risk difference (the difference in percent-point compared with the reference group); cRR, cumulative relative risk; CI, confidence interval in percentage; km, kilometer. * = statistical difference at p-value <0.05; ** = statistical difference at p-value <0.01; ***=statistical difference at p-value <0.001 | | | | | |
